# An investigation of tomosynthesis on the diagnostic efficacy of spot compression mammography

**DOI:** 10.1038/s41598-024-67332-y

**Published:** 2024-07-16

**Authors:** Ying Jiang, Lilin Yang, Rong Qian, Mingfang Li, Hong Pu, Aamer Rasheed Chughtai, Jinliang Hu, Weifang Kong

**Affiliations:** 1 Department of Radiology, Sichuan Provincial People’s Hospital, School of Medicine, University of Electronic Science and Technology of China, Chengdu, China; 2 Department of Radiology, Cleveland Clinic Health System, Cleveland, OH USA; 3 Department of Medical Records and Statistics, Sichuan Provincial People’s Hospital, School of Medicine, University of Electronic Science and Technology of China, Chengdu, China; 4 School of Computing and Artificial Intelligence, Southwest Jiaotong University, Chengdu, China

**Keywords:** Spot compression view, Tomosynthesis, Breast, Diagnostic efficacy, Mammography, Medical research, Oncology, Cancer, Breast cancer, Cancer imaging

## Abstract

To explore the diagnostic efficacy of tomosynthesis spot compression (TSC) compared with conventional spot compression (CSC) for ambiguous findings on full-field digital mammography (FFDM). In this retrospective study, 122 patients (including 108 patients with dense breasts) with ambiguous FFDM findings were imaged with both CSC and TSC. Two radiologists independently reviewed the images and evaluated lesions using the Breast Imaging Reporting and Data System. Pathology or at least a 1-year follow-up imaging was used as the reference standard. Diagnostic efficacies of CSC and TSC were compared, including area under the curve (AUC), accuracy, sensitivity, specificity, positive predictive value (PPV), and negative predictive value (NPV). The mean glandular dose was recorded and compared for TSC and CSC. Of the 122 patients, 63 had benign lesions and 59 had malignant lesions. For Reader 1, the following diagnostic efficacies of TSC were significantly higher than those of CSC: AUC (0.988 vs. 0.906, *P* = 0.001), accuracy (93.4% vs. 77.8%, *P* = 0.001), specificity (87.3% vs. 63.5%, *P* = 0.002), PPV (88.1% vs. 70.5%, P = 0.010), and NPV (100% vs. 90.9%, *P* = 0.029). For Reader 2, TSC showed higher AUC (0.949 vs. 0.909, *P* = 0.011) and accuracy (83.6% vs. 71.3%, *P* = 0.022) than CSC. The mean glandular dose of TSC was higher than that of CSC (1.85 ± 0.53 vs. 1.47 ± 0.58 mGy, *P* < 0.001) but remained within the safety limit. TSC provides better diagnostic efficacy with a slightly higher but tolerable radiation dose than CSC. Therefore, TSC may be a candidate modality for patients with ambiguous findings on FFDM.

## Introduction

Mammography is one of the primary and the most important imaging techniques in breast cancer screening and diagnosis^1,2^. With the widespread use of mammography, worldwide breast cancer mortality decreased from 18.4% in 2012 to 6.9% in 2020^3,4^. However, conventional mammography is limited by tissue superposition, and its false-negativity rate is approximately 15%^5^. To solve these problems, digital breast tomosynthesis (DBT) has been introduced. Tomosynthesis overcomes the problem of overlapping tissues by visualizing breast tissues slice by slice^6–8^. Previous studies^9–11^ confirm that DBT in standard (mediolateral-oblique and craniocaudal) views reduces the recall rate and improves the diagnostic accuracy in comparison with conventional full-field digital mammography (FFDM). However, it is still unclear whether tomosynthesis improves the diagnostic efficacy in special mammographic techniques, such as spot compression.

Since 1968, spot compression has been reported to aid FFDM in evaluating abnormalities or distinguishing real lesions from fake ones created by superimposed breast tissue^12–14^. It was a common method for further evaluation of suspicious lesions on FFDM. In recent years, tomosynthesis spot compression (TSC) has been introduced to clinical use; however, the diagnostic efficacy of TSC has yet to be investigated. The purpose of this study was to investigate the diagnostic efficacy of TSC, in comparison with that of conventional spot compression (CSC), for ambiguous findings on FFDM.

## Materials and methods

This study was approved by the ethics committee of Sichuan Provincial People's Hospital (Approval number: Lunshen (Research) 2023 No. 520). This was a retrospective study; thus, the requirement for informed consent was waived with approval from our institutional ethics committee.

### Patients

CSC and TSC were performed on patients with ambiguous findings on FFDM.

**Figure 1:**
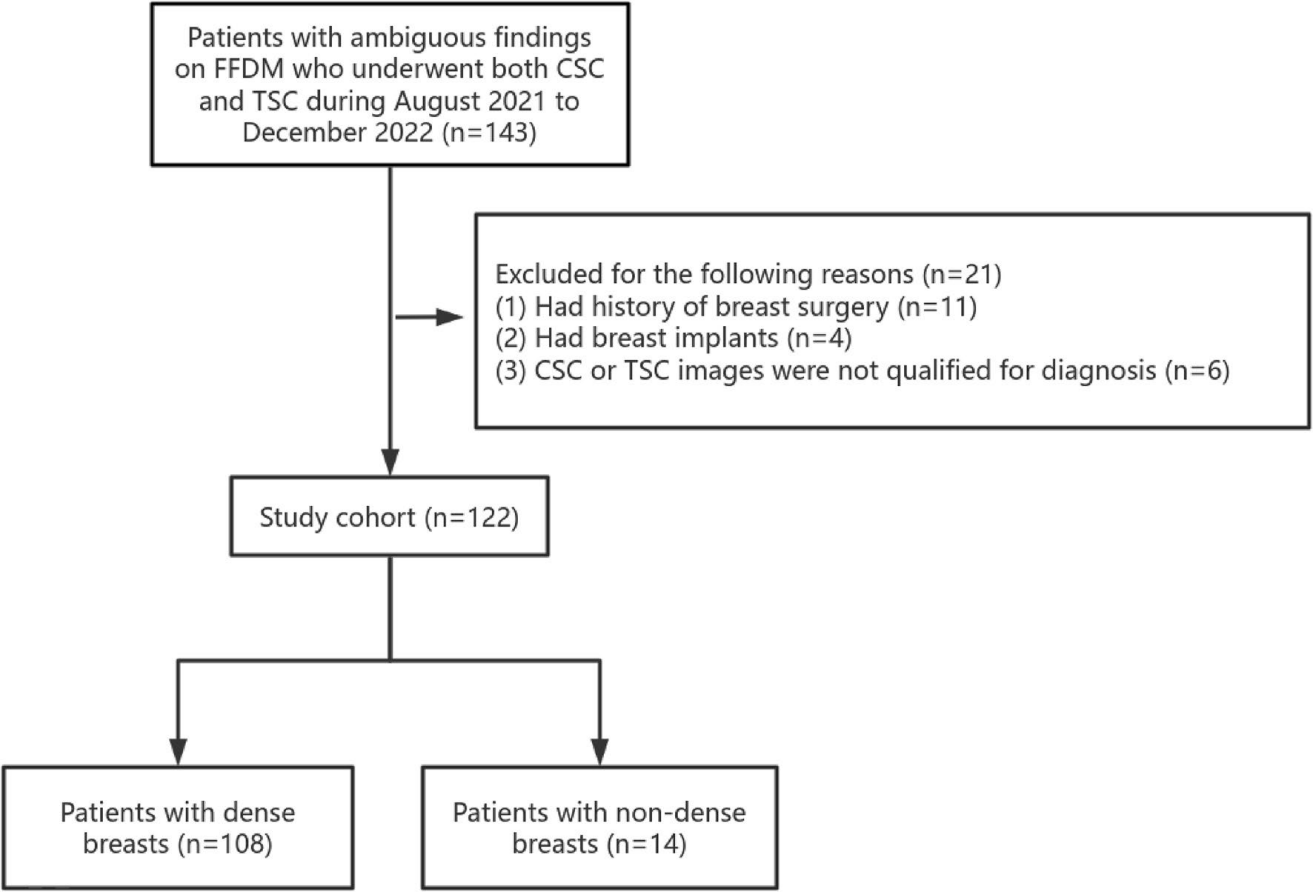
Flowchart showing the selection of patients.

Ambiguous findings on FFDM referred to findings that needed additional imaging studies to reach a more conclusive diagnosis, including findings assessed to be of Breast Imaging Reporting and Data System (BI-RADS) category 0, masses with obscured or indistinct margins, and suspicious architectural distortions and asymmetries. The images of 143 patients with ambiguous findings on FFDM from June 1, 2021 to December 31, 2022 were analyzed. We included the following: (1) inpatients and outpatients in our hospital with ambiguous findings on FFDM, with both TSC and CSC imaging data; and (2) patients with pathology results after TSC and CSC or those who were considered to have benign lesions based on at least 1 year of imaging follow-up. The following patients were excluded: (1) patients with a history of breast surgery; (2) patients with breast implants which may affect the images; and (3) patients with TSC or CSC images that were unqualified for diagnotic requirement (such as images wherein the lesion was not completely included in the field of view or those lost from the picture archiving and communication system [PACS]). Finally, 21 patients were excluded and 122 patients were included. The patient selection flowchart is shown in Fig. 1. The patients’ information, including age, initial symptoms, and histopathology results, were recorded from the PACS.

### CSC and TSC

Both CSC and TSC were performed using a commercial mammographic system (Selenia Dimensions; Hologic, MA, USA). Based on breast thickness and composition, optimal exposure parameters were calculated automatically using the automatic exposure control technique. Standard (mediolateral-oblique and craniocaudal) views of FFDM were first acquired for the patients. CSC and TSC were performed in those with ambiguous FFDM findings. The part of the breast where the lesion was identified was compressed using a spot compression paddle. CSC and TSC images were acquired with the same breast compression. In TSC, a series of low-dose projections were acquired with a 15° arc. The projections were subsequently combined to reconstruct 1-mm-thick tomosynthesis images. The CSC and TSC images were transmitted to a diagnostic review workstation for evaluation.

### Image analysis

Following the American College of Radiology BI-RADS atlas (5th edition)^15^, breast tissue, as seen on on FFDM images, was categorized into the following four types by a radiologist: (a) almost entirely fatty; (b) scattered areas of fibroglandular density; (c) heterogeneously dense; and (d) extremely dense. Types a and b were defined as non-dense breast tissues and types c and d, as dense breast tissues.

A senior radiologist (Reader 1) with 10 years of experience in breast imaging diagnosis and a junior radiologist (Reader 2) with 4 years of experience reviewed the CSC and TSC images independently. First, the lesions were evaluated and recorded on the CSC images. The assessment was then performed on the TSC images 3 months later. The two radiologists were blinded to the assessments made by each other, with no prior information on the patient or pathology. To reduce observation bias, the TSC images were displayed in different orders after 3 months. BI-RADS categories were used for the evaluation of the CSC and TSC images. Lesions in BI-RADS categories 2–4A were considered negative, and those in BI-RADS categories 4B–5 were considered positive. The diagnostic efficacy, including accuracy, sensitivity, specificity, positive predictive value (PPV), and negative predictive value (NPV), of CSC and TSC were compared among all the patients and among patients with dense and non-dense breasts by both readers separately. In addition, a 5-point Likert scale (1–5 corresponded to the level of suspicion of cancer: 1, not suspicious; 5, highly suspicious) was used to facilitate the receiver operating characteristic (ROC) analysis and to calculate the area under the ROC curve (AUC). The mean glandular dose (MGD) for TSC and CSC was recorded for each patient.

### Sample size calculation

According to the diagnostic performance of TSC reported by Deleau et al.^16^, the sensitivity and specificity of TSC ranged from 89 to 100% and 90–94%, respectively. We assumed the sensitivity and specificity for TSC to be equal to 95% and 92%, respectively. Based on these assumptions, using a two-sided binomial test, a total of 101 patients were required to achieve 90% efficacy for rejecting the null hypothesis. The target significance level was 0.05. A total of 122 patients were included, which provided > 90% power. The sample size was estimated using Power Analysis and Sample Size software (version 21.0.3; PASS).

### Statistical analysis

Kappa statistics were used to examine the intra-observer and inter-observer agreement between the two radiologists. Fisher's exact test or the χ^2^ test was used to compare the diagnostic efficacy parameters of CSC and TSC, as well as their diagnostic efficacy in dense breasts. The DeLong test was used to compare the AUC difference between CSC and TSC, and the Wilcoxon rank test was used to test the radiation dose difference between them. The radiation dose and patient age were summarized as the mean ± standard deviation. Statistical analysis was performed using SPSS 11.0 and MedCalc19.8 statistical software. A difference was considered statistically significant if the two-tailed *p*-value was < 0.05.

### Institutional review board statement

The study was conducted in accordance with the Declaration of Helsinki, and approved by the Ethics Committee of Sichuan Academy of Medical Science and Sichuan Provincial People’s Hospital (No. 2022118, 30 March 2022).

### Informed consent statement

Informed consent was obtained from the patients involved in the study.

## Results

### Patient sample

The patients’ ages ranged from 19 to 82 (47.73 ± 12.76) years. Among the 122 patients in this study, 59 had malignant lesions and 63 had benign lesions. The majority of the patients had dense breasts (108 patients) and 14 had non-dense breasts. On mammography, 59 patients had masses, 58 had asymmetries, and 5 had architectural distortions. The result of 23 patients were determined as benign based on over 1 year of follow-up imaging. The results of 94 patients were determined via core needle biopsy or excision. The benign lesions were mainly fibroadenoma and adenosis and the malignant lesions, invasive ductal carcinoma. Detailed information of these patients is shown in Table 1.

**Table 1:** Detailed information of the 122 patients included in this study.

	Number of patients	
	Malignant lesions	Benign lesions
Mammographic features		
Mass	39	20
Asymmetry	18	40
Architectural distortion	2	3
Calcification	0	0
Criteria for classification		
Core needle biopsy	35	36
Excision	24	4
Imaging follow-up		23
Histopathology results		
Malignant lesions		
Invasive ductal carcinoma	48	–
Ductal carcinoma in situ	7	–
Mucinous carcinoma	3	–
Papillary carcinoma	1	–
Benign lesions		
Fibroadenoma	–	19
Adenosis	–	16
Intraductal papilloma	–	2
Benign phyllodes tumor	–	2
Granulomatous mastitis	–	1
Unknown		23

### Inter-observer and intra-observer agreement

The inter-observer agreement for CSC and TSC was good (K = 0.698 [95% confidence interval < CI >  0.601–0.795] and K = 0.739 [95% CI 0.632–0.846], respectively). The intra-observer agreement of Reader 1 was good for both CSC (K = 0.735 [95% CI 0.625–0.845]) and TSC (K = 0.787 [95% CI 0.675–0.899]). The intra-observer agreement of Reader 2 was also good for both CSC (K = 0.654 [95% CI 0.519–0.789]) and TSC (K = 0.706 [95% CI 0.616–0.796]).

### Diagnostic efficacy

**Figure 2:**
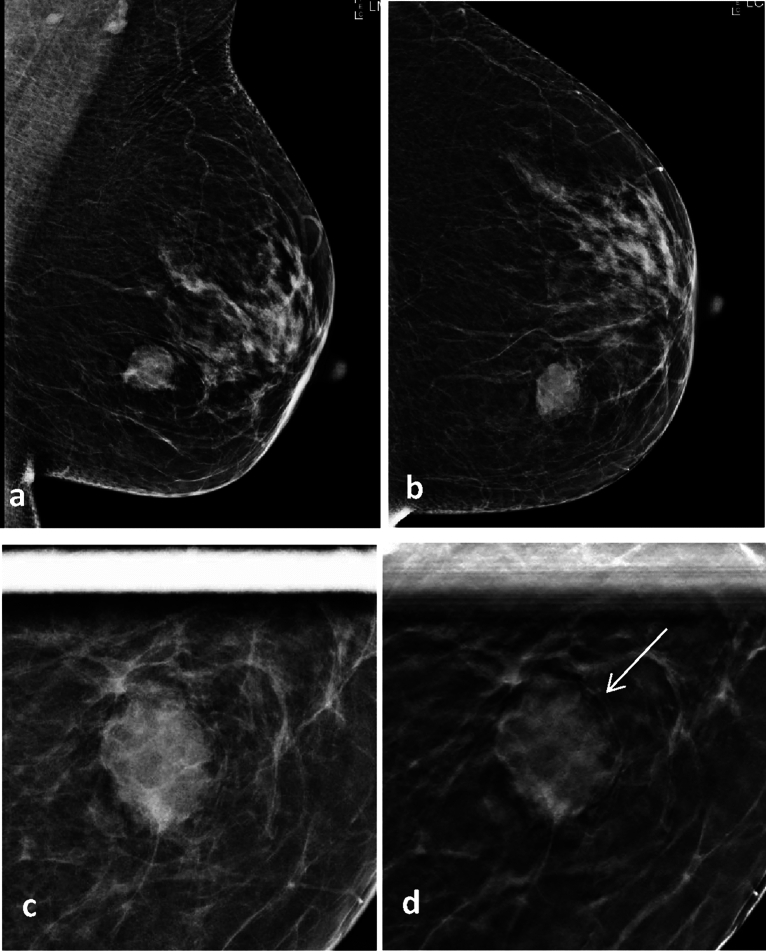
A 69-year-old woman with fibroadenoma confirmed via core needle biopsy. (**a**,**b**) Full-field digital mammography shows a mass in the lower inner quadrant of the left breast; however, the margin of the mass is partially obscured, requiring further evaluation. (**c**) Conventional spot compression view shows a high density mass with an indistinct margin, categorized as Breast Imaging Reporting and Data System (BI-RADS) 4B. (**d**) The spot compression tomosynthesis image shows an associated benign feature, the “Halo sign” (arrow), which is a low-density ring of compressed adipose tissue around the lesion. The mass was categorized as BI-RADS 3.

**Figure 3:**
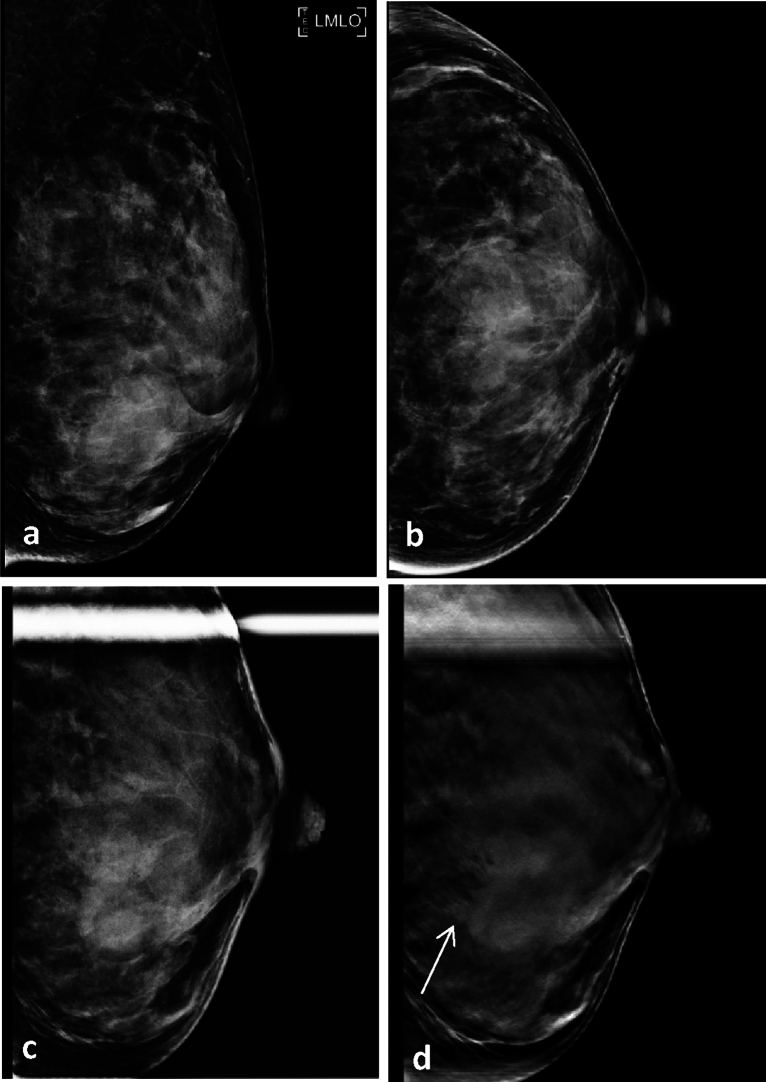
A 39-year-old woman with invasive ductal carcinoma confirmed via excision biopsy. (**a**,**b**) Full-field digital mammography shows a vague mass in the lower center of the left breast. (**c**) Conventional spot compression view shows a high-density mass with an obscured margin, categorized as Breast Imaging Reporting and Data System (BI-RADS) 4A. (**d**) Spot compression tomosynthesis clearly shows a spiculated margin (arrow); thus, the lesion was categorized as BI-RADS 4C.

Among the 63 cases of benign masses, 29 were assigned lower BI-RADS categories on TSC images compared with those on CSC images by Reader 1, whereas 9 were similarly assigned by Reader 2. An example of a benign mass categorized as BI-RADS 4B on CSC images and categorized as BI-RADS 3 on TSC images is shown in Fig. 2. Among the 59 cases of malignant cases, 19 were assigned higher BI-RADS categories on TSC images compared with those on CSC images by Reader 1, whereas 12 were similarly assigned by Reader 2. An example of a malignant mass categorized as BI-RADS 4A on CSC images and as BI-RADS 4C on TSC images is shown in Fig. 3. The BI-RADS categories assigned by both readers for the lesions on TSC and CSC images are shown in Table 2.

**Table 2:** BI-RADS categories of lesions assigned by the two readers using CSC and TSC* images.

BI-RADS category	Reader 1				Reader 2			
	Benign		Malignant		Benign		Malignant	
	CSC	TSC	CSC	TSC	CSC	TSC	CSC	TSC
2	11	19	1	0	16	20	1	1
3	20	23	0	0	16	17	1	1
4	32	21	58	59	31	26	57	57
4A	19	16	8	2	22	17	8	1
4B	13	5	17	8	9	9	19	19
4C	0	0	27	42	0	0	22	27
5	0	0	6	7	0	0	8	10

*BI-RADS Breast Imaging Reporting and Data System; *CSC* spot compression view; *TSC* spot compression tomosynthesis.

For Reader 1, the diagnostic performance using TSC images was significantly better in terms of accuracy (93.4% vs. 77.8%, *P* = 0.001), specificity (87.3% vs. 63.5%, *P* = 0.002), PPV (88.1% vs. 70.5%, *P* = 0.010), and NPV (100% vs. 90.9%, *P* = 0.029), compared with that using CSC images. Reader 2 showed better accuracy using TSC images versus CSC images (83.6% vs. 71.3%, *P* = 0.022). Other data on diagnostic efficacy also showed equivalent or higher efficacy using TSC images versus CSC imaging; however, the differences were not significant.

Among the patients with dense breasts, the following variables were significantly higher for Reader 1 when using TSC data versus CSC data: accuracy (95.4% vs. 78.7%, *P* < 0.001), specificity (91.7% vs. 66.7%, *P* = 0.001), and PPV (90.6% vs. 69.2%, *P* = 0.005). Reader 2 showed a higher accuracy (84.3% vs. 70.4%, *P* = 0.015) using TSC data versus CSC data. The sample size of patients with non-dense breasts was too small to conduct meaningful statistical analysis. The diagnostic efficacies of both readers using CSC and TSC data are shown in Table 3.

**Table 3:** Diagnostic efficacy using CSC and TSC* images.

	All patients			Patients with dense breasts		
	CSC	TSC	*P*	CSC	TSC	*P*
Reader 1						
AUC	0.906	0.988	0.001	0.909	0.992	0.002
Accuracy	77.8% (95/122)	93.4% (114/122)	0.001	78.7% (85/108)	95.4% (103/108)	< 0.001
Sensitivity	93.2% (55/59)	100% (59/59)	0.119	93.8% (45/48)	100% (48/48)	0.242
Specificity	63.5% (40/63)	87.3% (55/63)	0.002	66.7% (40/60)	91.7% (55/60)	0.001
PPV	70.5% (55/78)	88.1% (59/67)	0.010	69.2% (45/65)	90.6% (48/63)	0.005
NPV	90.9% (40/44)	100% (55/55)	0.029	93.0% (40/43)	100% (55/55)	0.081
Reader 2						
AUC	0.909	0.949	0.011	0.907	0.951	0.011
Accuracy	71.3% (87/122)	83.6% (102/122)	0.022	70.4% (76/108)	84.3% (91/108)	0.015
Sensitivity	88.1% (52/59)	96.6% (57/59)	0.163	85.4% (41/48)	95.8% (46/48)	0.160
Specificity	55.6% (35/63)	71.4% (45/63)	0.064	58.3% (35/60)	75.0% (45/60)	0.053
PPV	65.0% (52/80)	76.0% (57/75)	0.134	62.1% (41/66)	75.4% (46/61)	0.107
NPV	83.3% (35/42)	95.7% (45/47)	0.078	93.0% (40/43)	95.7% (45/47)	0.078

*CSC spot compression view; *TSC* spot compression tomosynthesis; *AUC* area under the receiver operating characteristic curve; *PPV* positive predictive value; *NPV* negative predictive value.

**Figure 4:**
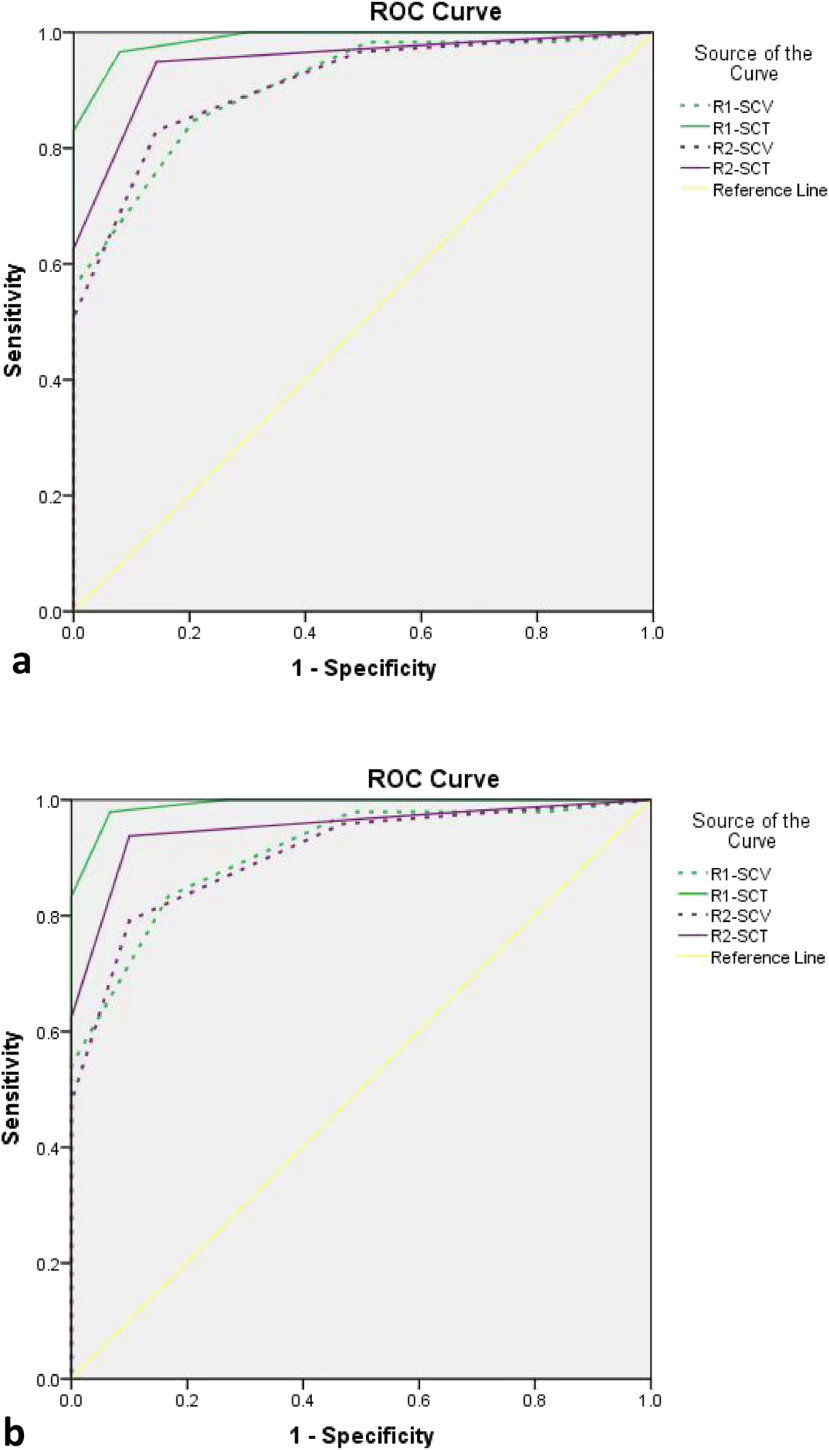
Comparison of ROC curves for both readers using CSC and TSC images of (**a**) all patients and (**b**) patients with dense breasts. *R1: Reader with 10 years of experience; R2: Reader with 4 years of experience; CSC: spot compression view; TSC: spot compression tomosynthesis; ROC: receiver operating characteristic.

Based on the 5-point Likert scale, the AUC when using TSC data was higher than that when using CSC data for both Reader 1 (0.988 vs. 0.906, *P* = 0.001) and Reader 2 (0.949 vs. 0.909, *P* = 0.011). For dense breasts, the AUC using TSC data was still higher than that when using CSC data for both Reader 1 (0.992 vs. 0.909, *P* = 0.002) and Reader 2 (0.951 vs. 0.907, *P* = 0.011). The ROC curves of both readers for CSC and TSC data from all the patients and those with dense breasts are shown in Fig. 4.

### Radiation dose

The radiation dose of TSC was significantly higher than that of CSC. The MGD of CSC and TSC were 1.47 ± 0.58 mGy (0.54–3.70 mGy; median MGD, 1.36 mGy) and 1.85 ± 0.53 mGy (0.97–4.40 mGy; median MGD, 1.85 mGy), respectively (*P* < 0.001).

## Discussion

DBT in standard views and CSC are both clinically available for patients who have ambiguous findings on FFDM, such as vague masses, architectural distortions, and asymmetries^11–14^. DBT in standard views not only shows more detailed structures of lesions but also helps to localize one-view findings. Therefore, tomosynthesis improves the clinical accuracy of mammography by increasing both sensitivity and specificity^17,18^. Meanwhile, CSC also helps to improve the clinical accuracy by reducing glandular overlaps^12,13^. TSC combines the advantages of tomosynthesis and spot compression and is therefore expected to have better diagnostic efficiency. The results of this study show that, the diagnostic efficacy is significantly improved when using CSC data compared with that when using TSC data, especially for patients with dense breasts. This indicates that TSC may be a better choice than CSC for evaluating patients with ambiguous findings on FFDM.

We studied cases that were assigned different BI-RADS categories on CSC and TSC images. For cases with malignant lesions, both readers assigned higher BI-RADS categories on TSC images compared with that on CSC images. TSC can show indistinct or spiculated margins of masses that are obscured on CSC or architectural distortions around asymmetrical lesions which are not obvious on CSC. Most cases of benign lesions were assigned lower BI-RADS categories on TSC images compared with that on CSC images by both readers. Analysis showed that the margin of the circumscribed mass was better shown on TSC images than on CSC images. Another reason was that TSC revealed associated benign features, such as the “Halo sign” of the mass, which was a low-density ring of compressed adipose tissue around the mass. The advantage of TSC may come from its ability of resolving tissue overlap and better showing the lesion margin and surrounding structures.

It was observed that three cases of benign lesions were assigned a higher BI-RADS category on TSC images compared with that on CSC images by the less experienced reader. All three cases had asymmetries, indicating that, for less experienced readers, some asymmetries are difficult to identify. Previous studies^19,20^ suggest that training is important for less experienced radiologists to improve the learning curve and fully take advantage of tomosynthesis.

Meanwhile, our results show that, for an experienced reader (with 10 years of experience), the diagnostic performance using TSC images was significantly better than that when using CSC images, including a higher AUC, accuracy, specificity, PPV, and NPV. For a less experienced reader (with 4 years of experience), the diagnostic efficacy was still improved when using TSC images versus CSC images, with a higher AUC and accuracy. Although the use of TSC improved the diagnostic efficacy of both readers, a certain level of experience may be required to use its full potential. This indicates that standardized training for radiologists is essential for the application of TSC in clinical practice.

The study cohort mostly comprised patients with dense breasts. The data collected from the patients with dense breasts showed that the improvement in diagnostic efficacy when using TSC data versus CSC data is significant. However, the sample size of patients with non-dense breasts was too small to conduct meaningful statistical analysis; thus, the diagnostic efficacy of TSC for non-dense breasts is still unclear, making further investigation with a larger sample size necessary.

Our study shows that TSC has an approximately 20%-higher dose than does CSC; however, it is still within the safety limit. Compared with the significant improvement in diagnostic performance, the slight and tolerable increase in dose is well-justified.

Our research results are clinically significant for the following reasons: (1) the study preliminarily shows that tomosynthesis can still exert its advantages on the basis of CSC, broaden the application scenarios of tomosynthesis, and verify the feasibility and radiation dose of TSC; (2) it can help optimize the examination process, as it proves that TSC can be a better choice, compared with CSC, for evaluating patients with ambiguous findings on FFDM and those with dense breasts, especially in the areas where mammography is the primary examination technique for breast disease; and (3) Tagliafico et al.^11^ found that the diagnostic accuracy of DBT in standard views is at least equal to that of CSC. Because our results show that the diagnostic efficiency of TSC is better than that of CSC, we speculate that the efficiency of TSC is also better than that of DBT in standard views, though this needs to be verified in our next study.

The limitations of this study include its retrospective design and small sample size. We hope to conduct additional investigations with a larger number of patients. Moreover, just a few patients in our center underwent both DBT in standard views and TSC; therefore, TSC and DBT in standard views could not be directly compared. We aim to further investigate this with a larger volume of data. In addition, all the images were obtained using one mammographic device from a single manufacturer. A future multi-center study may provide better results.

In conclusion, TSC provides better diagnostic efficacy with a slightly increased dose, compared with CSC. It is a promising alternative to CSC for examining patients with ambiguous findings on FFDM.

## Data Availability

The datasets generated during and/or analyzed during the current study are not publicly available but are available from the corresponding author upon reasonable request.
